# Machine learning-enhanced insights into sphingolipid-based prognostication: revealing the immunological landscape and predictive proficiency for immunomotherapy and chemotherapy responses in pancreatic carcinoma

**DOI:** 10.3389/fmolb.2023.1284623

**Published:** 2023-10-31

**Authors:** Ting Shi, Minmin Li, Yabin Yu

**Affiliations:** Department of Hepatobiliary Surgery, The Affiliated Huaian No 1 People’s Hospital of Nanjing Medical University, Huaian, China

**Keywords:** pancreatic carcinoma, machine learning, sphingolipid, immunotherapy response, prediction

## Abstract

**Background:** With a poor prognosis for affected individuals, pancreatic adenocarcinoma (PAAD) is known as a complicated and diverse illness. Immunocytes have become essential elements in the development of PAAD. Notably, sphingolipid metabolism has a dual function in the development of tumors and the invasion of the immune system. Despite these implications, research on the predictive ability of sphingolipid variables for PAAD prognosis is strikingly lacking, and it is yet unclear how they can affect PAAD immunotherapy and targeted pharmacotherapy.

**Methods:** The investigation process included SPG detection while also being pertinent to the prognosis for PAAD. Both the analytical capability of CIBERSORT and the prognostic capability of the pRRophetic R package were used to evaluate the immunological environments of the various HCC subtypes. In addition, CCK-8 experiments on PAAD cell lines were carried out to confirm the accuracy of drug sensitivity estimates. The results of these trials, which also evaluated cell survival and migratory patterns, confirmed the usefulness of sphingolipid-associated genes (SPGs).

**Results:** As a result of this thorough investigation, 32 SPGs were identified, each of which had a measurable influence on the dynamics of overall survival. This collection of genes served as the conceptual framework for the development of a prognostic model, which was carefully assembled from 10 chosen genes. It should be noted that this grouping of patients into cohorts with high and low risk was a sign of different immune profiles and therapy responses. The increased abundance of SPGs was identified as a possible sign of inadequate responses to immune-based treatment approaches. The careful CCK-8 testing carried out on PAAD cell lines was of the highest importance for providing clear confirmation of drug sensitivity estimates.

**Conclusion:** The significance of Sphingolipid metabolism in the complex web of PAAD development is brought home by this study. The novel risk model, built on the complexity of sphingolipid-associated genes, advances our understanding of PAAD and offers doctors a powerful tool for developing personalised treatment plans that are specifically suited to the unique characteristics of each patient.

## 1 Introduction

Pancreatic adenocarcinoma is recognized as one of the most prevalent forms of primary malignancies globally (Huang C. et al., 2023; Huang X. et al., 2023). Notably, the complex interaction of inflammatory cascades plays a crucial role in the development of PAAD. This cancer develops from dysplastic nodules and progresses through a range of histopathological phases, each with unique molecular and cellular characteristics (Chi et al., 2023a). Despite sincere efforts to remove the tumor surgically (Li C. et al., 2023), PAAD patients typically experience poor prognosis results (Zhang B. et al., 2022; Zhang et al., 2023a; Cui et al., 2023). This bleak reality can be attributed to the PAAD’s substantial intratumoral and interpatient heterogeneity, which inexorably encourages the development of drug resistance events and disease recurrence (Baek and Lee, 2020; Chi et al., 2022a; Wen et al., 2023; Yu et al., 2023). Checkpoint inhibitor immunotherapy is a cutting-edge cancer treatment (Zhang L. et al., 2022; Su et al., 2022). Checkpoint inhibitors have emerged as pivotal agents in the management of PAAD, colorectal malignancies, and various other neoplastic diseases (Zhang P. et al., 2022; Zhang et al., 2023b; Zhang L. et al., 2023). Furthermore, PAAD’s complex interaction with the immunological environment has gained attention from researchers. The tailored RNA neoantigen vaccines treatment strategy for pancreatic cancer has received attention from recent clinical studies (Rojas et al., 2023).

However, the need for accurate identification and validation of reliable prognostic biomarkers poses a significant obstacle to the efficient translation of checkpoint immunotherapy to the PAAD scenario. This need stems from the urgent need to improve therapeutic strategies and, ultimately, patient outcomes. Therefore, there is still a need for novel biomolecular markers that predict patient prognoses, and their discovery might help PAAD enter a new age of individualized medication.

Sphingolipids, essential structural elements of cellular membranes, play a crucial and multidimensional function in the complex control of a variety of biological processes (Chi et al., 2022b). These include and go beyond important processes including cellular development, proliferation, directed migration, invasion, and even the metastatic cascade, particularly in the setting of malignancy (Ogretmen, 2018; Yuan et al., 2022a). As secondary messengers, they go beyond their structural function to exert control on cellular differentiation, senescence, programmed cell death, and general growth dynamics. Sphingomyelin, ceramide, sphingosine-1-phosphate, a substance reviewed in its name, and glycosphingolipids are among the fundamental components supporting the field of sphingolipids (Xiao et al., 2019; Sasset and Di Lorenzo, 2022). Dynamic changes in the complex sphingolipid biosynthesis pathway have the potential to delicately modify a variety of signaling cascades, dramatically affecting the course of carcinogenesis either as a promoter or an inhibitor (Guri et al., 2017; Muthusamy et al., 2020; Qi et al., 2021; Thayyullathil et al., 2021). In recent years, research on the role of sphingolipids in oncology has garnered significant attention, particularly in the context of tumor immunotherapy and chemotherapy (Zhang X. et al., 2023; Machy et al., 2023; Su et al., 2023). Studies have indicated that certain sphingolipids can modulate the immunogenicity of tumor cells, rendering them more susceptible to recognition and elimination by the immune system (Kozbor, 2010; Zhong et al., 2022). Furthermore, the alteration of sphingolipid composition on the membranes of immune cells has been demonstrated to enhance their activity, thereby augmenting their anti-tumor potential (Kue et al., 2012). These investigations provide robust support for the development of novel tumor immunotherapies. Sphingolipids also play a pivotal role in chemotherapy (Brachtendorf et al., 2019). Some studies have suggested that sphingolipids can influence the sensitivity of tumor cells to chemotherapeutic agents (Sousa et al., 2023). By regulating the levels of sphingolipids on cell membranes, it is possible to either increase or decrease the resistance of tumor cells to these drugs (Chiu et al., 2022). Recent studies have shown a significant correlation between some sphingolipid cohort members and the development of PAAD, providing predictive information about the course of the illness (Horejsi et al., 2023; Wilson et al., 2023). Despite the well-established importance of sphingolipids in the field of PAAD, there has been a noticeable lack of thorough investigations into the latent predictive value held by the SPGs, or short for sphingolipid orchestration genes, genes. A deeper and more thorough understanding of these genetic components has the potential to improve patient survival rates and increase response to treatment plans.

The evolution of bioinformatics has found extensive utility in the identification of biological markers and the diagnosis of diseases (Jin et al., 2021a; Jin et al., 2021b; Yan et al., 2021; Liu G. et al., 2022; Li et al., 2022). Nevertheless, exploration of genes associated with lipid bilayers remains notably limited. By using SPGs derived from the TCGA-PAAD cohort, our research aims to develop a reliable predictive model. Following the integration of this genetic data with essential clinicopathological factors, a nomogram is created that is designed to improve prognosis accuracy and provide customized therapeutic care methods. We meticulously validated the proposed nomogram using rigorous studies, such as time-dependent Receiver Operating Characteristic (ROC) and Decision Curve Analysis (DCA), to determine its clinical prognostic usefulness. These analytical methods allow for a thorough assessment of the nomogram’s performance, demonstrating its reliability for clinical settings. Our empirical findings suggest that sphingolipid-associated genes have the latent ability to predict the future course of individuals with PAAD. Additionally, the found genes provide up fresh possibilities as empirically confirmed indicators, hence increasing the pool of targets for targeted therapy approaches.

## 2 Materials and methods

### 2.1 Data procurement

Four samples of normal pancreatic tissue and 179 cases of PAAD from the TCGA-PAAD cohort’s transcriptomic data were obtained from the TCGA repository (Zhai et al., 2020; Wang X. et al., 2022). Additionally, 179 PAAD tumor patients’ clinical data were gathered, a total of 179 PAAD patients were further filtered based on the completeness of survival time and age, as well as their survival status (Leek et al., 2012).

### 2.2 The acquisition of genes related to sphingolipids

InnateDB stands as a publicly accessible repository, encompassing genes, proteins, experimentally validated interactions, and signaling pathways pertinent to the innate immune responses to microbial infections in humans. This repository augments the scope of innate immune interaction networks by amalgamating known interactions and pathways from major public databases with meticulously curated datasets into a centralized resource. Through the use of the InnateDB gateway (https://www.innatedb.com/index.jsp), 97 SPGs were assembled (Breuer et al., 2013).

### 2.3 The LASSO regression test

In the context of this study, univariate Cox regression analysis was used to identify 32 SPGs that showed associations with PAAD patients’ survival rates. The “glmnet” R package (Song et al., 2022a) then made it easier to do LASSO regression analysis (Chi et al., 2023b; Ren et al., 2023), where the parameter was found using tenfold cross-validation (Zhao et al., 2023a). A multivariate Cox regression model was ultimately used to identify a group of ten key genes (Guan et al., 2023). Ten SPGs were used to create a risk signature by utilizing the best lambda scores and coefficients. Riskscore = SPTLC3*0.4708+STS*0.1031+KDSR*0.2274+SPHK1*0.1951+ARSJ*0.1008-ARSG*0.9889-CERS3*2.8795-CERS4*0.1164-SPHK2*0.3553-SMPD2*0.0189.

### 2.4 Evaluation of immune cell invasion

Utilizing the CIBERSORT and ssGSEA R scripts (Newman et al., 2015; Zhao S. et al., 2022; Song et al., 2022b; Ren et al., 2022) allowed for the measurement of infiltrating immune cell numbers. Immune cell type scores were calculated for individual samples using the CIBERSORT method (Zhao et al., 2023b). As a result, the predicted immune cell type scores were used to determine the scores for each sample. Furthermore, Spearman correlation analysis was used to look at the relationship between immune cell profiles and risk scores. The immune cell profiles of PAAD patients were used to inform the ssGSEA approach, which was then used to identify various risk categories for different people (Chi et al., 2023c).

### 2.5 Predicting the therapeutic response to chemotherapy

The R package “pRRophetic” serves as a valuable tool for the application of gene expression data in predicting drug responses (Shen et al., 2022; Wang Z. et al., 2023). Its underlying principle relies on training models using established drug response data and subsequently employing these models to map new gene expression data onto predictions of drug responses. This approach contributes significantly to the realm of personalized medicine and the formulation of tailored pharmaceutical treatment strategies (Yuan et al., 2022b). The “pRRophetic” R program was used to calculate the IC50 of small molecule medicines.

### 2.6 Cell culture

The PAAD cell lines AsPC-1 and Panc-10.05, which were grown at 37 °C in a 5% CO2 atmosphere, were supplied by the ATCC firm. The culture media used was PRIM 1640 from Thermo Scientific, with 15% fetal bovine serum from Gibco as an addition (Song et al., 2021; Zhai et al., 2023).

### 2.7 Assay for cell viability

The Cell Count Kit-8 (Dojindo, Japan) was utilized to determine the cell viability of AsPC-1 and Panc-10.05 cells. Cells were cultured at 37 °C with 5% CO2 for a period of 2 h after the addition of 10 L of CCK-8 reagent to each well. The subsequent measurement of optical density (OD) values at 450 nm ensued (Cai et al., 2022; Zhang Y. et al., 2023). Assessment of migration capability for AsPC-1 and Panc-10.05 cells was performed after a seeding interval of 48 h. Following that, the values of optical density (OD) at 450 nm were measured. After a 48-h seeding period, the capacity of AsPC-1 and Panc-10.05 cells to migrate was evaluated.

### 2.8 Statistical analysis

R version 4.1.3 was used to conduct all data analysis. The Student’s t-test was used to variables with a normal distribution, and Pearson’s correlation coefficient was used to evaluate inter-variable relationships. Statistical analysis of cellular experiments was conducted using GraphPad Prism 8 and SPSS Statistics v.27 software, with statistical analysis performed utilizing t-tests. The thresholds for statistical significance were *p* < 0.05*, *p* < 0.01**, and *p* < 0.001***, respectively.

## 3 Results

### 3.1 Development of the sphingolipid gene signature

The acquisition of a thorough set of 97 genes strongly associated with sphingolipid metabolism, painstakingly extracted from the prestigious InnateDB platform, served as the basis for our investigation. We obtained the necessary dataset from the TCGA database to provide a solid basis for our following studies as our investigation narrowed in on the complex landscape of PAAD. The “limma” R program was used to carefully go through the transcriptome data, finding genes within the sphingolipid pathway that had distinct expression patterns across the PAAD tumor samples and their corresponding surrounding normal tissues.

**FIGURE 1:**
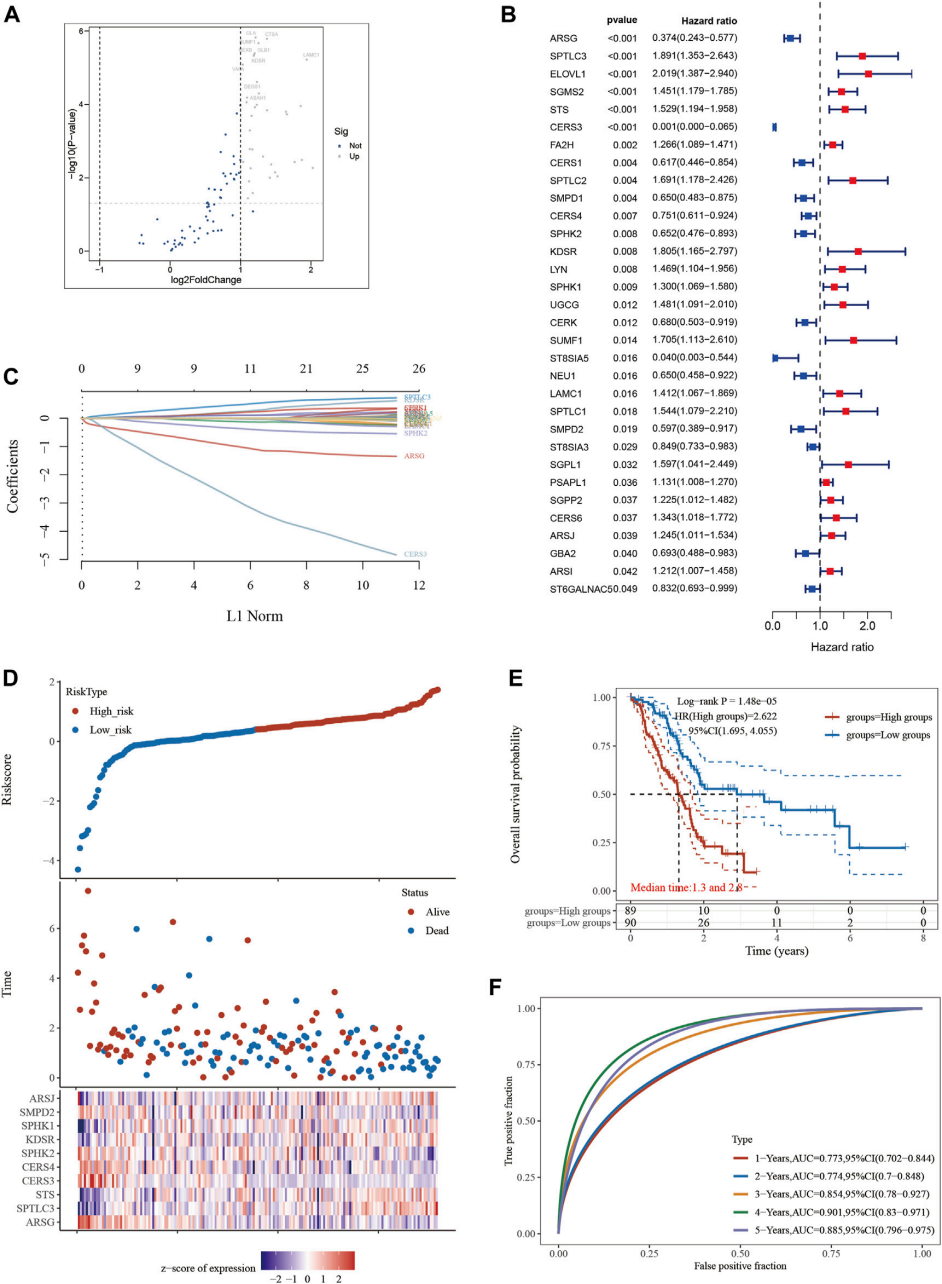
Constructing a prognostic model based on sphingolipid-related genes in PAAD. **(A)** Differential gene screening was conducted to identify SPGs associated with PAAD. **(B)** 13 genes of prognostic significance, which we refer to as SPGs, were identified from the differential gene screening analysis. These SPGs demonstrated an association with survival in PAAD patients. **(C)** Utilizing the Lasso method, a prognostic model was constructed based on the identified SPGs. **(D)** The risk scores, survival status, and expression levels of the top 10-SPGs were plotted to visualize the distribution of prognostic risk. **(E)** Kaplan-Meier (KM) analysis was performed to further investigate the prognostic significance of the 10-SPGs in different PAAD subtypes. **(F)** The predictive efficiency of the prognostic model was evaluated using ROC analysis.

A thoughtful statistical methodology was used to identify a group of 35 SPGs that were associated to sphingolipids and showed notable expression difference (Figure 1A). The investigation of these SPGs went beyond simple expression alterations to include the critically important area of patient survival. Utilizing a comprehensive strategy, we evaluated the complex interactions between SPGs and the survival outcomes of PAAD patients using the analytical power of the “survival” and “survminer” R packages. A group of 32 SPGs that were closely linked to patient survival developed from the initial 35 SPGs (Figure 1B). Notably, a sizeable group of SPGs deviated from this general pattern and were negatively linked with good prognosis. These contrasts served as the foundation for the future creation of a PAAD prognostication prediction model. A model with possible therapeutic value was revealed by a careful Lasso analysis built around the 32 survival-associated SPGs (Figure 1C). It was crucial to confirm the predicted precision of this model. The model’s predictive accuracy was evaluated using time-dependent ROC curve analysis over many temporal horizons, including 1, 2, 3, and 5 years, all of which highlighted its dependability (Figure 1F). By using a dichotomization technique to divide the cohort of 179 PAAD patients into high- and low-risk categories based on median riskscore, the practical implications of this model were further highlighted. This line of demarcation clearly showed a perceptible survival disparity, with the high-risk group showing noticeably reduced survival (Figure 1E), where median survival durations of 1.3 and 2.8 years were recorded, respectively. Last but not least, we created a heatmap depicting the expression patterns of the top 10 SPGs across multiple riskscore groups to provide a visual summary of the expression trends within the context of riskscore stratification (Figure 1D).

### 3.2 SPG expression differentiation across subtypes

**FIGURE 2:**
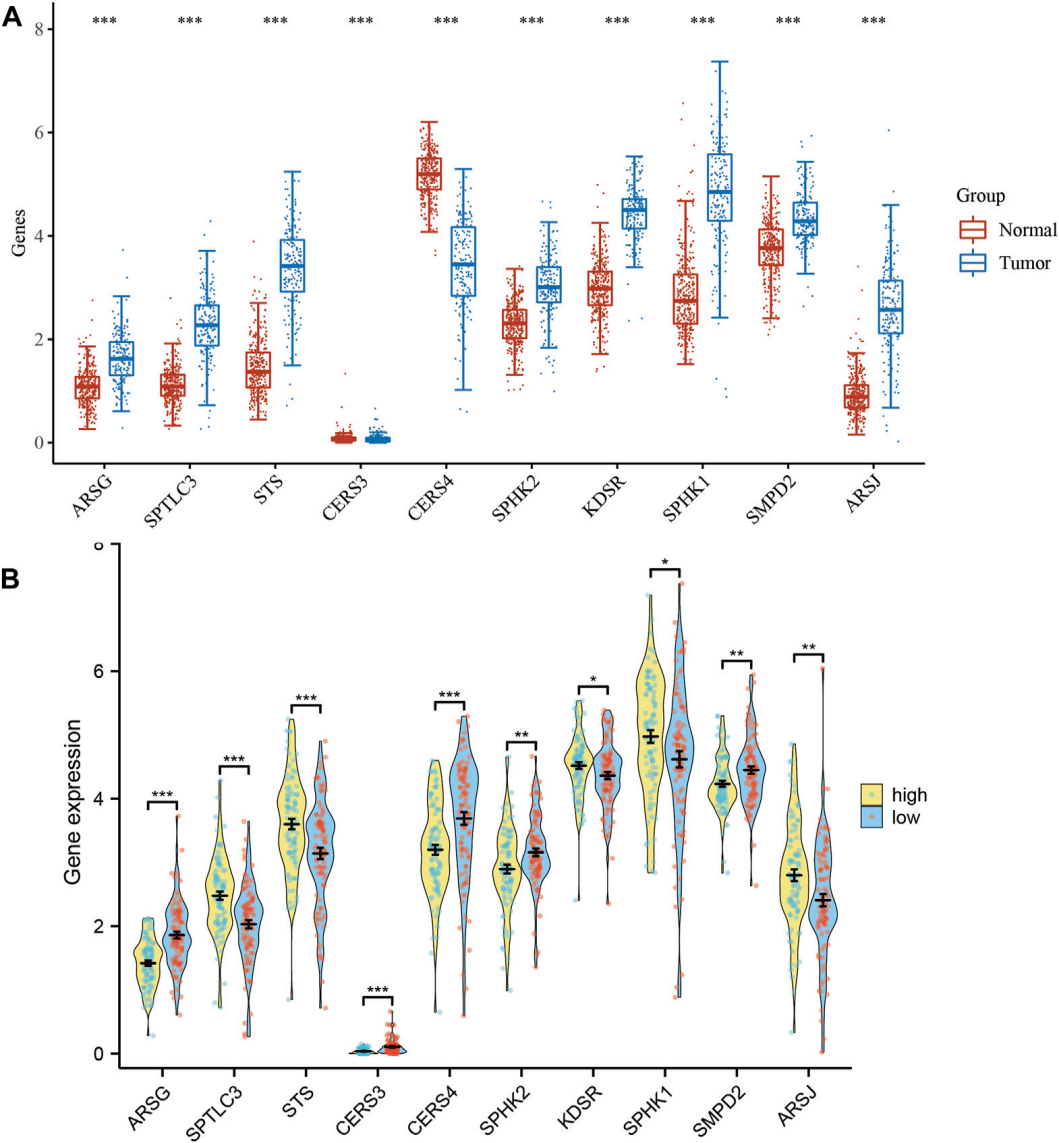
Expression levels of 10-SPGs. **(A)** Expression levels of 10-SPGs in PAAD tumor tissues and adjacent tissues. **(B)** Expression levels of 10-SPGs in PAAD risk subgroups. (*p < 0.05, **p < 0.01, ***p < 0.001).

We conducted a thorough analysis of the transcriptional patterns of the ten SPGs in both normal and tumorous tissues using mRNA expression levels as a quantitative measure (Figure 2A). When compared to their nearby non-neoplastic counterparts, all 10 SPGs were evidently expressed differently in tumor tissues (*p* < 0.05), with SPHK1 particularly showing the highest level of expression. We subsequently examined the expression levels of these 10 SPGs among high-risk and low-risk subgroups in an effort to understand the underlying biological consequences of these observed patterns. We noticed a fascinating discrepancy between the ten SPGs in this subgroup’s expression pattern and the trend shown in Figure 1A, which is striking (Figure 2B). In order to get a deeper understanding, we used Kaplan-Meier survival curves to outline the relationship between each important SPG gene and the prognosis of PAAD patients. All 10 SPGs were shown to be statistically significantly correlated with patient prognosis according to this thorough research (*p* < 0.05). This highlights both their clinical importance and their potential to serve as prognostic indicators in PAAD, in addition to their clinical significance.

### 3.3 GO and KEGG enrichment analysis

**FIGURE 3:**
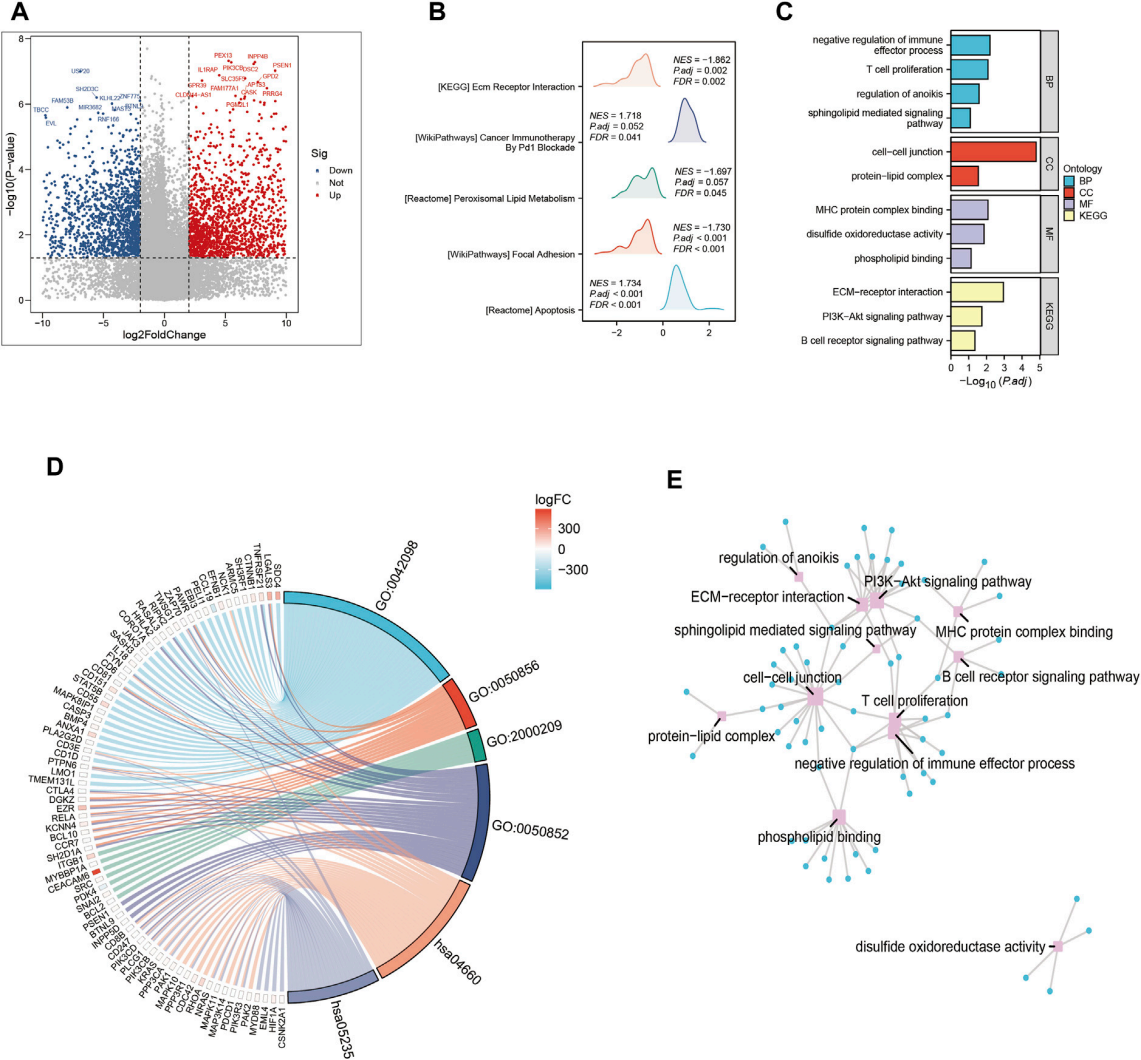
Gene ontology (GO) and Kyoto Encyclopedia of Genes and Genomes (KEGG) analysis **(A)** Volcano map screening for differential genes. **(B)** Mountain map showing the enriched KEGG pathway. **(C–E)** GO enrichment analysis.

In this part, we systematically examined the effects of several signaling pathway activations on the complex dynamics driving tumor cell proliferation and development, as well as their complex interaction within the tumor microenvironment. A systematic comparison of gene expression levels was carried out in order to identify genes with differential expression patterns that could be used to identify cohorts with high-risk and low-risk profiles (Figure 3A). A constellation of pathways with notable enrichment found among the group of individuals who showed increased vulnerability to PAAD. These pathways, which are individually highlighted by their significant enrichment coefficients (Figure 3B), include but are not limited to those of major relevance, such as Cancer Immunotherapy. As we dug further, the interesting details of the high-risk subgroup’s transcriptional landscape were revealed by our investigation of Gene Ontology enrichment. Conspicuously elevated were the processes that control cell-cell junctions, which are essential for cellular adhesion and communication. On the other hand, there was a noticeable downregulation of pathways involved in the negative regulation of immune effectors. This subtle modulation, seen in Figure 3C, points to a well planned interaction between tumor cells and their immune environment. Additionally, a concentrated investigation into the genes that differed the most between the high-risk and low-risk groupings made it possible to identify crucial Gene Ontology pathways. The connections between these pathways and the underlying genetics of risk stratification were clearly shown in (Figures 3D,E), shedding light on proposed molecular mechanisms controlling various risk traits.

### 3.4 Patients with PAAD have varying degrees of immunological infiltration

**FIGURE 4:**
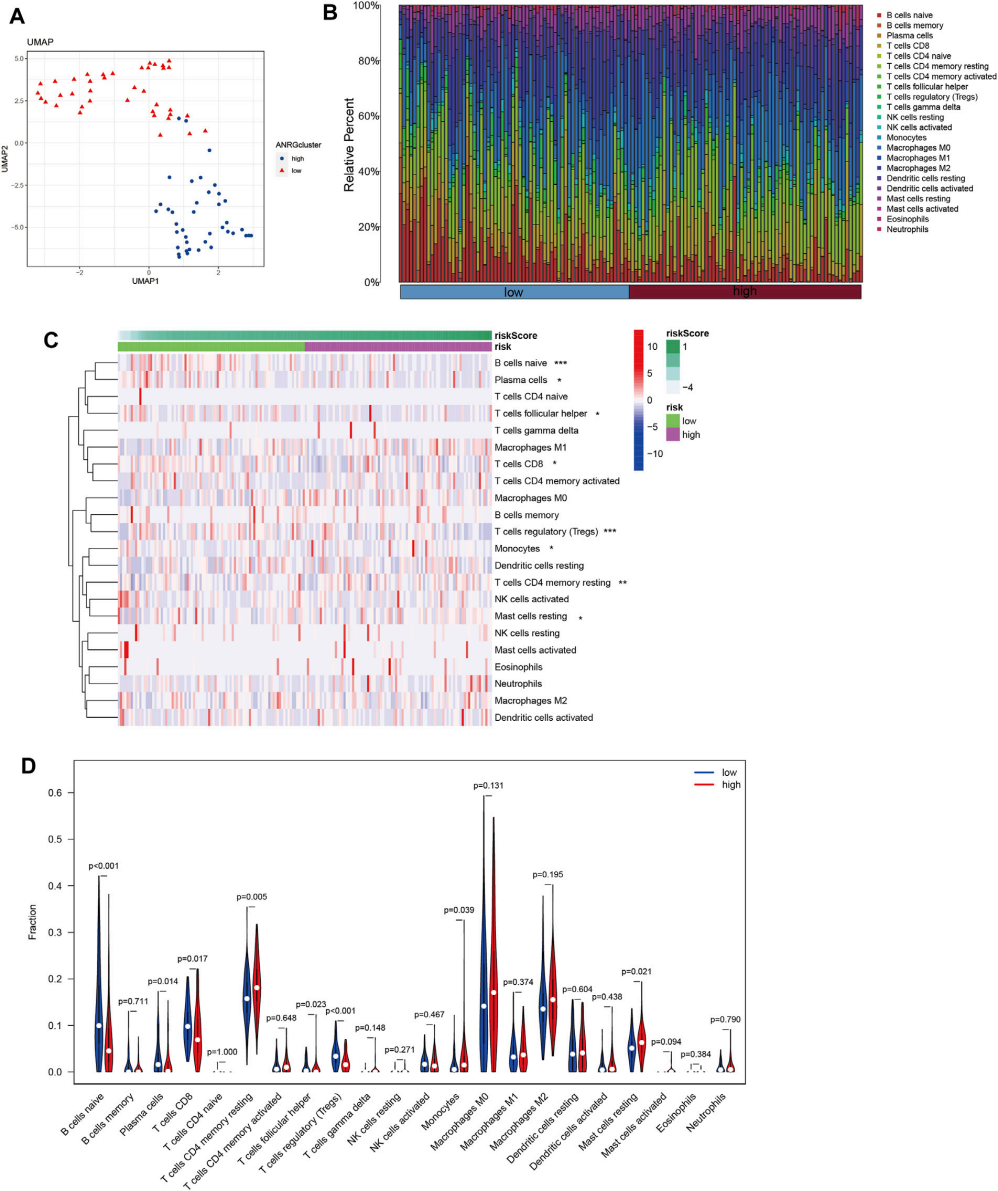
Identify immune landscape of PAAD based on sphingolipid signature. **(A)** UMAP demonstrates different immune profiles among PAAD subgroups. **(B)** Proportion of immune cells in PAAD tissues. **(C, D)** Differences in immune infiltration between PAAD subgroups.

**FIGURE 5:**
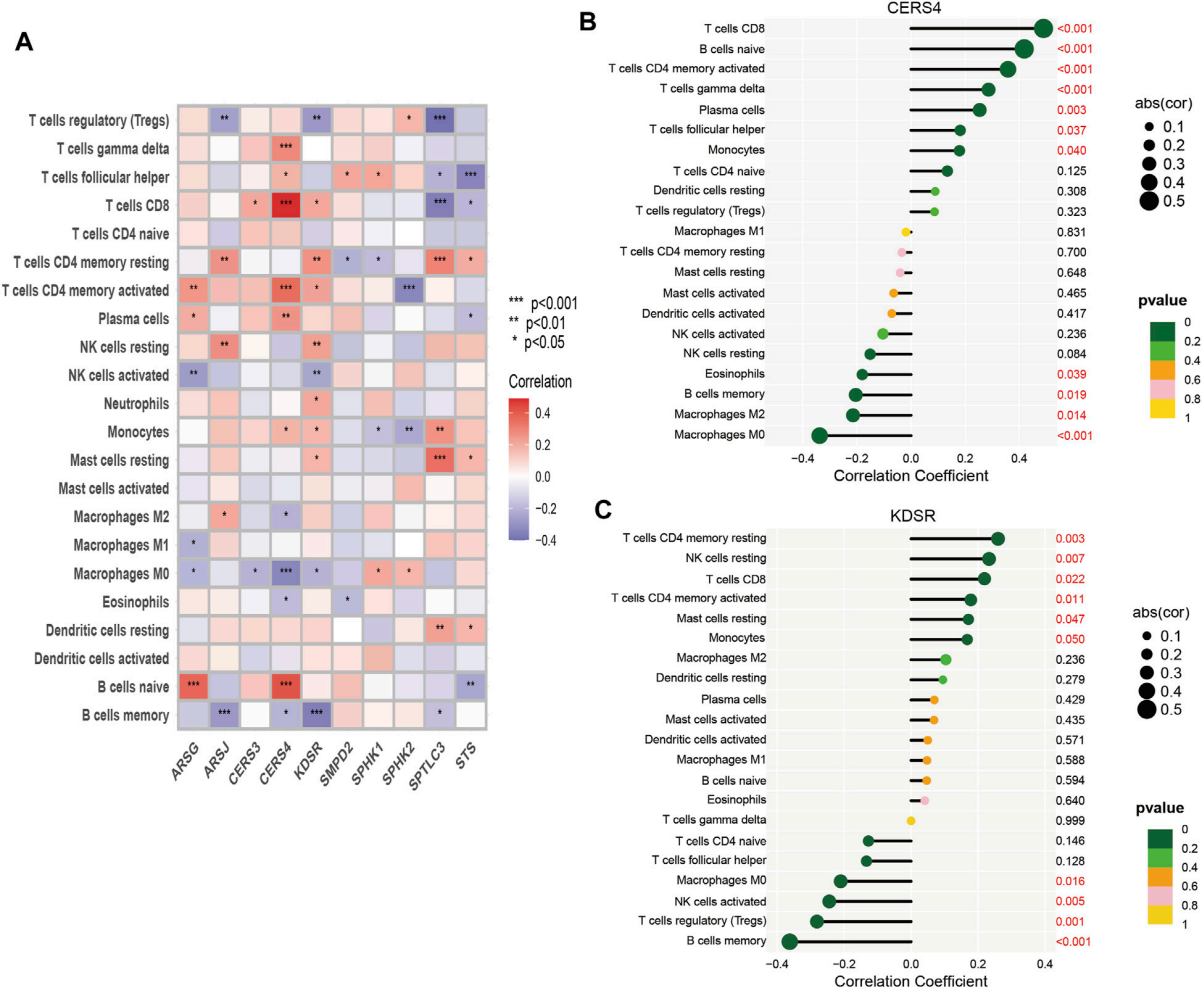
Correlation between immune cells and 10-SPGS. **(A)** Heatmap was used to show the correlation between immune cells and 10-sphingolipid genes (10-SPGs). **(B, C)** Bar plots were used to illustrate the relationship between CERS4 and KDSR with immune cell infiltration.

Tumor growth is heavily influenced by the microenvironment of a tumor, particularly the immune system. Tumor cells can avoid immune surveillance when the immune system is not working correctly (Zhao Y. et al., 2022; Chi et al., 2022c). We performed dimensionality reduction and clustering using the Lasso method on a particular collection of 10-SPGs selected for PAAD patients. Our study’ results confirm the effectiveness of these 10-SPGs in differentiating between PAAD patients with different risk propensities (Figure 4A). The complicated patterns of immune infiltration among PAAD patients, stratified by various prognostic factors, were further investigated (Figures 4B–D). The ordering of risk score values, which reflects the proportionate distribution of various immune cell subtypes, in ascending order is an interesting finding (Figure 4B). Importantly, our research reveals that among PAAD patients categorized as high-risk, there is a noticeable infiltration of certain immune cell types, including CD4 memory resting T-cell and dormant Mast cells. The CD8 T-cell and Tregs within this subgroup, on the other hand, show a noticeable drop (Figures 4C,D). Comparing high-risk PAAD patients to those with low risk reveals that there is less immune cell infiltration, which may be a sign of a weaker response to ICB treatments. Our analysis goes further and looks at the relationship between the 10-SPGs and immune cell populations (Figure 5) in order to further understand this discovery. Multiple immune cell types’ expression patterns of 3 SPGs were shown to be significantly correlated (Figure 5A). Notably, the infiltration of CD8 T-cell is strongly positively correlated with CERS3 and KDSR (Figures 5B,C). This link raises the possibility of a relationship between the immune milieu and the expression of these sphingolipid genes in PAAD patients.

### 3.5 Analysis of the relationship between immunotherapeutic effectiveness and SPG expression

**FIGURE 6:**
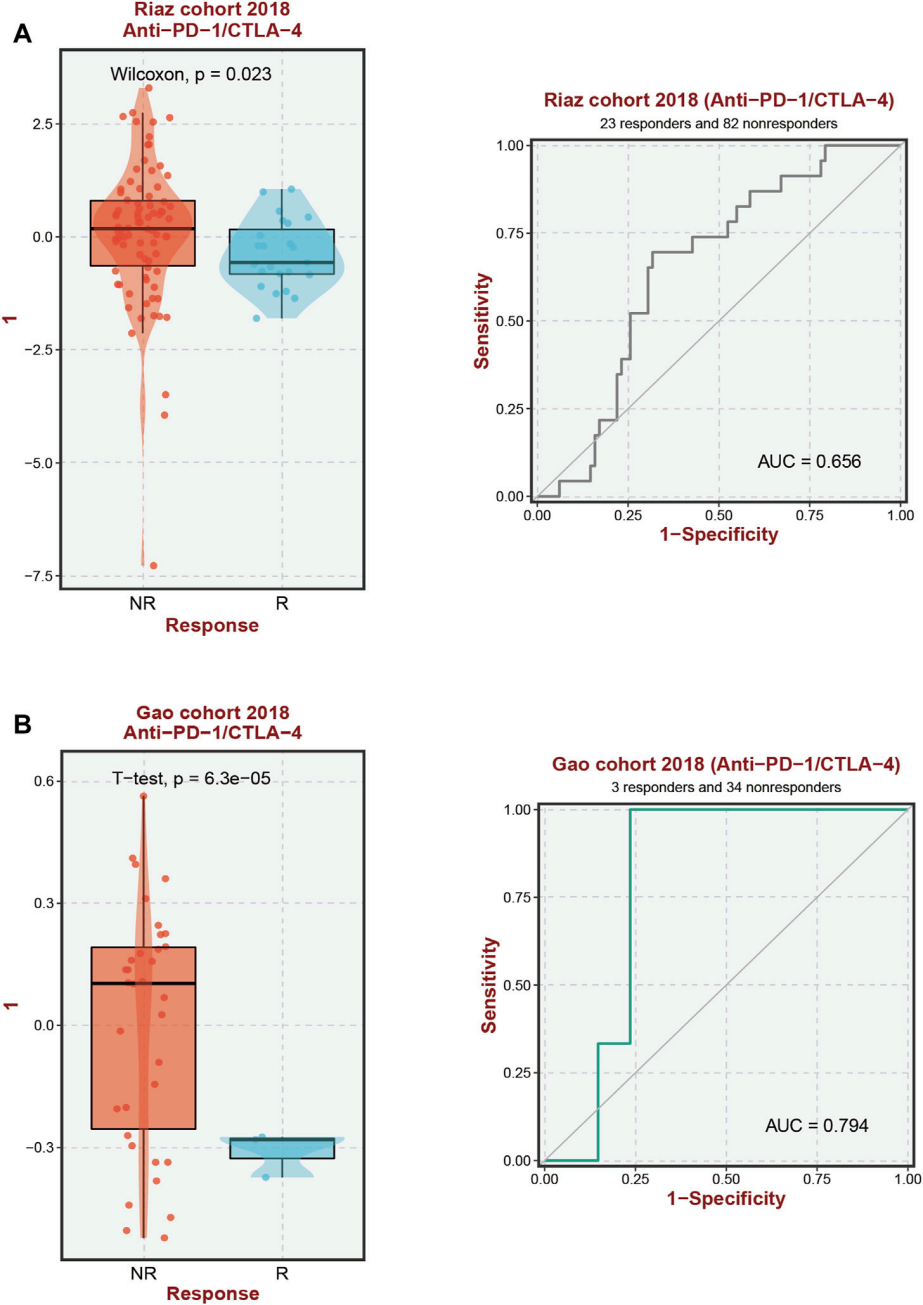
Immunotherapy response prediction. **(A, B)** Prediction of immune therapy response to anti-PD-1/CTLA-4 treatment in PAAD patients based on 10-SPGS.

**FIGURE 7:**
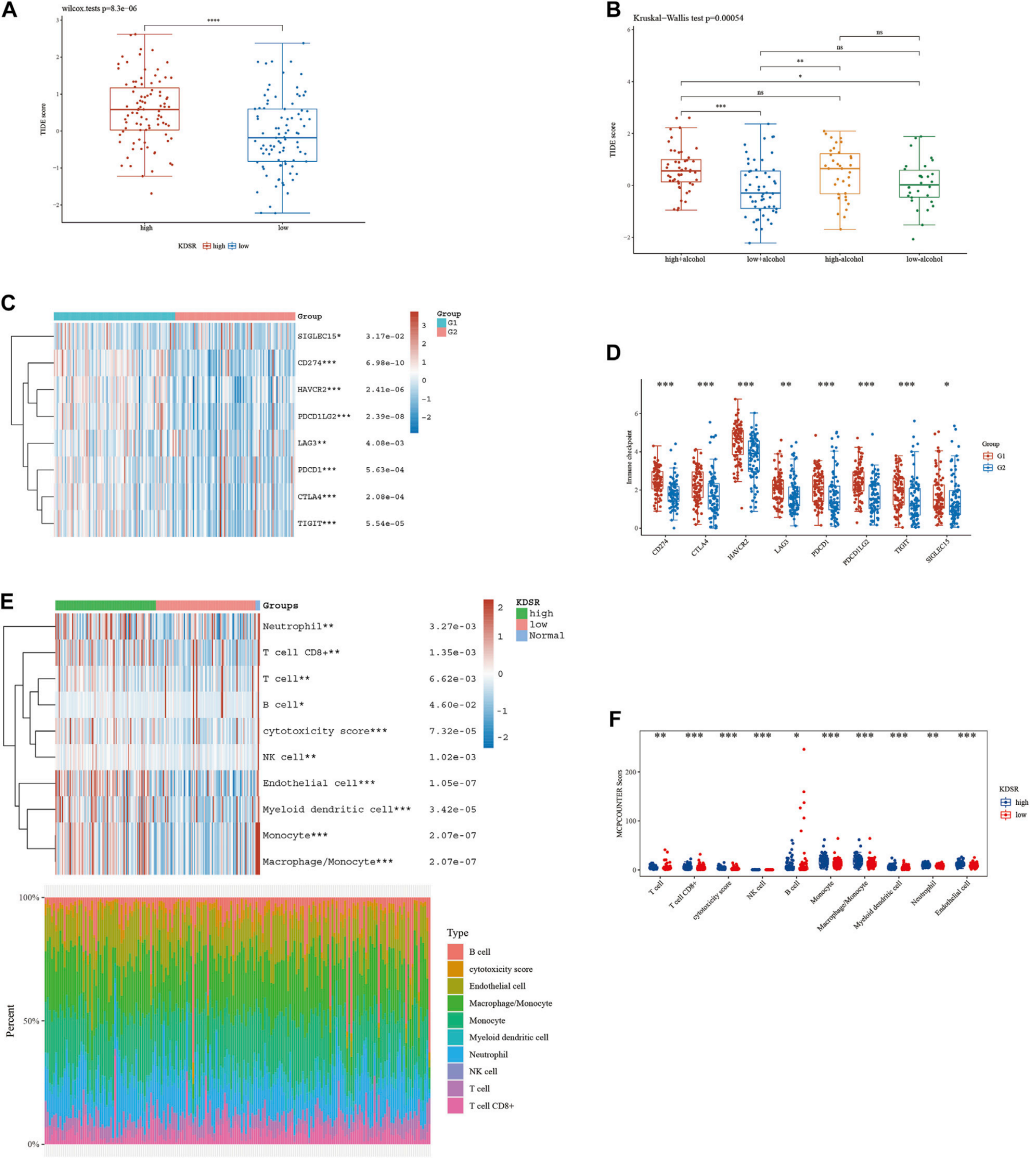
The level of immune checkpoint in PAAD subtypes. **(A)** The TIDE score between KDSR subgroups. **(B)** Alcohol does not affect the effectiveness of immunotherapy. **(C, D)** There are differences in the expression of immune checkpoint markers between the high-risk and low-risk groups of PAAD. **(E, F)** CIBERSORT analysis revealed differences in immune infiltration between the subgroups.

According to our analysis’s preliminary findings, there are observable differences in the immunological milieu across cohorts at high and low risk. Particularly, there is a notable decrease in the infiltration of both Tregs and CD8 T-cell within the high-risk population. A milieu that is immunologically quiescent is produced by the convergence of these changed immune components, and this microenvironment in turn has differential effects on the effectiveness of the two different immunotherapeutic methods under review. It is significant to note that individuals with decreased 10-SPG cluster expression levels have a propensity for good responses to both PD-L1 and PD-1 blocking therapy (Figure 6). It is interesting to note that the 10-SPG cluster also shows promise as a prognosis tool, providing the capacity to prognosticate the accuracy of ICB therapy in the population of patients with PAAD (Figures 6A,B). When we focus on the molecular environment, a striking indication that predicts a poorer prognosis for PAAD patients is the increase in KDSR expression inside high-risk PAAD cancer tissues. This poor prognosis is thought to be caused by the concurrent increase in immune response activity (Figure 1B, 2B), a theory supported by the elevated immune response shown in this subgroup (Figure 7A). We used a comprehensive strategy to try to understand the differences in ICB responses between high-risk and low-risk PAAD patients. We connected KDSR expression levels with the complex dynamics of alcohol exposure using the Tumor Immune Dysfunction and Exclusion (TIDE) algorithm (Figure 7B). Surprisingly, this combination of factors showed that increased KDSR expression is still a reliable indicator of elevated immune response scores, unaffected by the subtleties of alcohol use. We looked further into the molecular factors after being intrigued by the effect of KDSR on immune response score. We also looked at the immune checkpoint expression patterns within the group that had been divided up by various SPG risk ratings. Surprisingly, a recurrent pattern showed that CD274, and CTLA4 were among the critical immunological checkpoints that were significantly upregulated in the high KDSR expression subgroup (Figures 7C,D). Finally, we utilized the computational capability of Cibersort analysis to fully assess the immunological milieu. Using this analytical method, we were able to compare the immune cell infiltration rates between the normal, low-risk, and high-risk PAAD tissue specimens (Figures 7E,F). The results of this research highlighted clear differences in immune cell infiltration across the various groups, providing better understandings of the complex interactions between immunological profiles and disease risk.

### 3.6 Forecasting and verification of drug responsiveness

**FIGURE 8:**
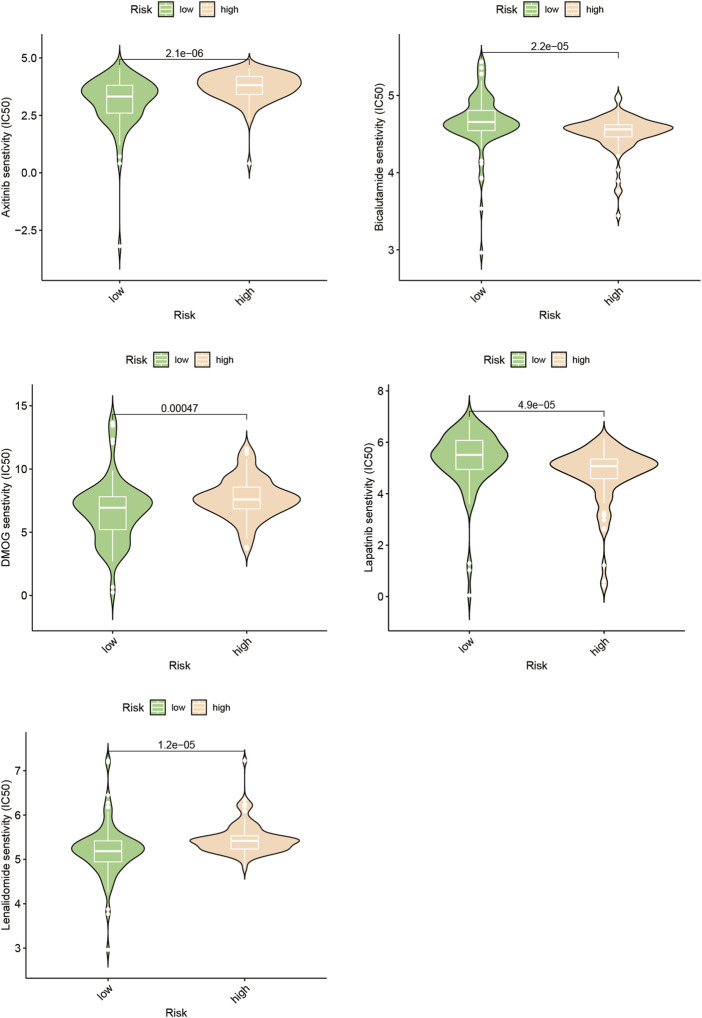
Drug sensitivity prediction.

Poor outcomes in cancer are significantly influenced by the recurrence of the disease through metastasis (Chen et al., 2019). We investigated the differences in drug response to chemotherapeutic drugs among subgroups distinguished by increased and lowered risk scores in the search for customized treatments for PAAD patients. Our study compared the IC50 values of five different chemotherapeutic drugs in the high-risk and low-risk clusters, two clearly defined subpopulations with different risk ratings (Figure 8, Supplementary Table S1). The findings of our investigation revealed significant differences in the IC50 measurements for a few drugs, including Axitinib. This finding suggests that those with high riskscores might potentially show increased vulnerability to this particular chemotherapy regimen.

**FIGURE 9:**
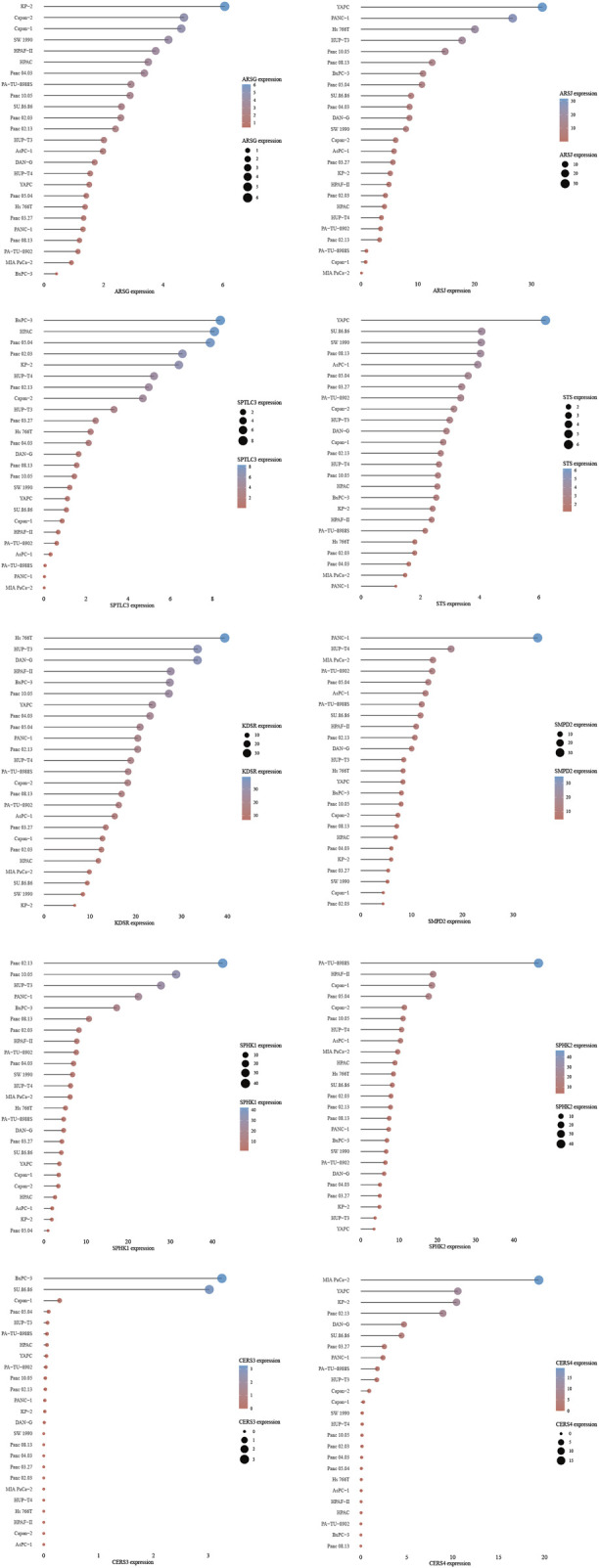
SPGs expression levels in pancreatic cancer cell lines.

**FIGURE 10:**
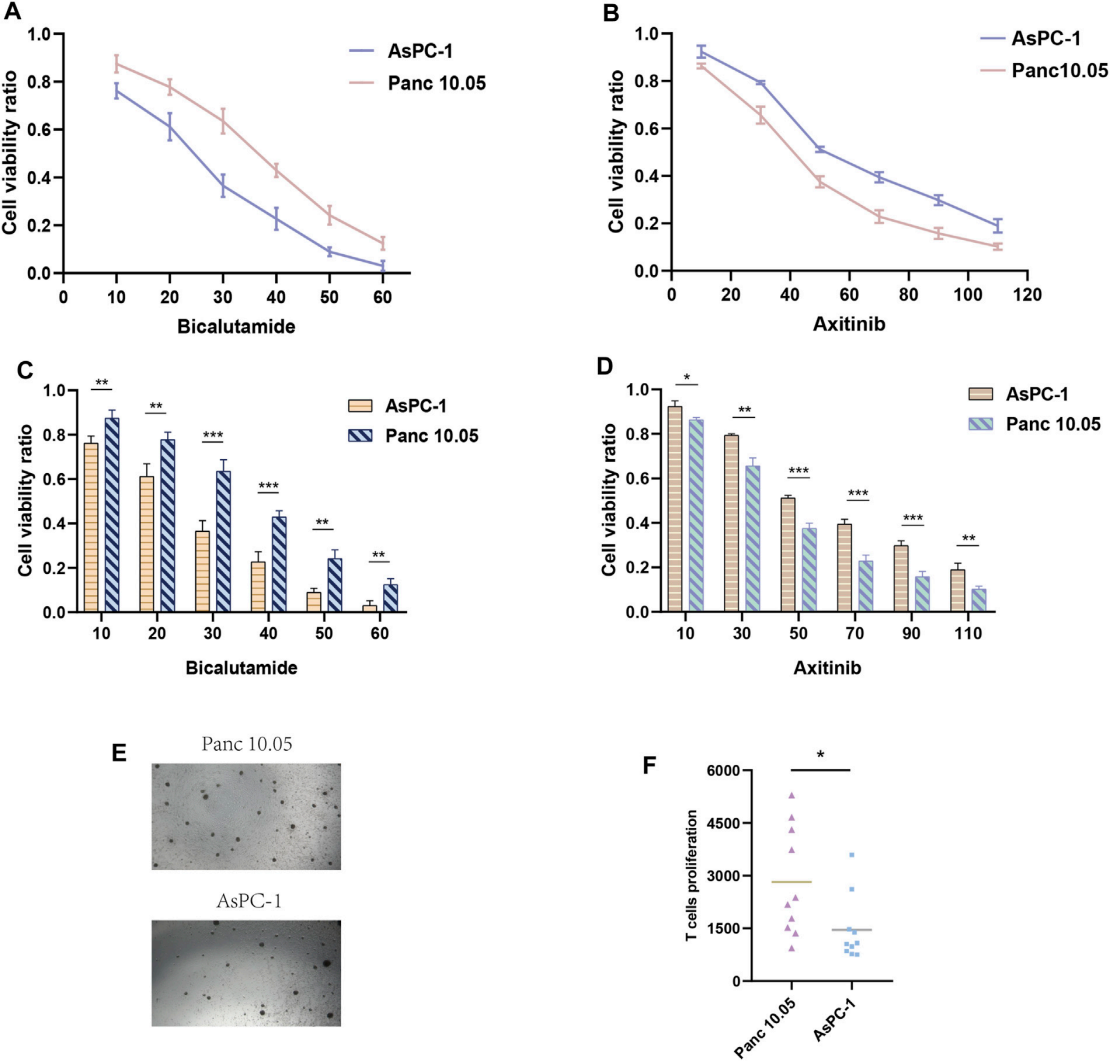
Drug sensitivity experiments. **(A, C)** The CCK-8 assay revealed the cytotoxic effects of Bicalutamide on Panc-10.05 and AsPC-1 cells at different concentrations. **(B, D)** The CCK-8 assay revealed the cytotoxic effects of Axitinib on Panc-10.05 and AsPC-1 cells at different concentrations. **(E, F)** T-cell proliferation induced by pancreatic cancer cells.

We evaluated other PAAD cell lines’ risk scores based on the mRNA expression of each SPGs (Figure 9), in order to support our findings and provide another layer of validation. The Panc 10.05 and AsPC-1 cell lines were chosen to adequately reflect the two dichotomous groups of PAAD patients, defined by their high-risk and low-risk scores, respectively, in the context of drug responsiveness tests. Our CCK-8 test findings showed that AsPC-1 cells, a representative of the low-risk population, displayed a more pronounced sensitivity to Bicalutamide as compared to their Panc 10.05 counterparts, which was consistent with the predictions about drug responsiveness (Figure 10A). In contrast, Panc 10.05 cells were more sensitive to Axitinib compared with AsPC-1 cells (Figure 10B). This agreement supports the idea that Bicalutamide and Axitinib could develop into a good candidate for targeted, precision-oriented treatment approaches for PAAD patients (Figures 9C,D).

### 3.7 The capacity of diverse-risk PAAD cell lines to induce T-cell proliferation

The work that came before it highlights the elevated immunoreactivity that high-risk PAAD patients show in comparison to their low-risk peers. We suggest that identifying high-risk PAAD cells based on genes associated to sphingolipids might result in a more robust stimulation of T-cell maturation. To test this hypothesis, we co-cultured T-cell with Panc 10.05 cells, which had a high riskscore, and AsPC-1 cells, which had a low risk score, for a period of 14 days. We then observed the level of T-cell activation and proliferation. Our findings demonstrate a significantly higher rate of T-cell proliferation in Panc 10.05 cells with a high risk score compared to AsPC-1 cells (**Figures 10E,F**), suggesting a potential relationship between Sphingolipid-related genes and the expression of surface-mutated proteins and, in turn, inducing a higher response to T-cell proliferation.

## 4 Discussion

The onset of diseases is frequently influenced by a multitude of factors, encompassing hormones (Luo et al., 2021), metabolic byproducts (Liu J. et al., 2023; Zhang et al., 2023f), inflammatory states (Xiao et al., 2020; Ni et al., 2021; Zhang et al., 2023g). Notably, certain determinants can precipitate the genesis of neoplasms. For instance, epigenetics is intricately intertwined with tumorigenesis (Wang J. et al., 2022; Zhang et al., 2023h), while microorganisms and immune cells play pivotal roles in shaping the course of neoplastic developments (Gong et al., 2023; Xia et al., 2023). Furthermore, the migratory propensity of tumor cells is closely associated with adverse prognoses and recurrent occurrences (Li Z. et al., 2023). A dangerous cancer known as pancreatic adenocarcinoma poses a serious threat to human life (Yuan et al., 2022c; Liu X. et al., 2023; Zhang et al., 2023i). The improvement of prognosis is a challenging endeavor because to the complex molecular pathways underlying PAAD. Single pharmacological therapies or single-targeted pathway interventions are ineffective in improving the prognosis of PAAD (Halbrook et al., 2023; Qi et al., 2023; Vahabi et al., 2023). As a result, using a wide variety of genes to build prognostic models turns out to be a more effective tactic. However, there are currently not enough effective biomarkers designed to achieve this goal. The need for further biomarkers is urgently needed to improve prognostic model accuracy and enable preventative actions against PAAD in its early stages.

Sphingolipids, a family of lipids, have a significant role in the maintenance of structural integrity and the sensitive management of membrane fluidity, as previously noted in studies (Zhang et al., 2023a). Cellular membranes are complex assemblages of various lipids. In particular, tumor cells’ metabolic needs are essential for their continued growth and survival inside the intricate tumor microenvironment (Soltani et al., 2021). Sphingolipids, recognized as bioactive molecules in the lipid repertory, have a diverse role in a variety of key cellular functions, acting not only as structural elements but also as essential mediators in cell signaling pathways (Gault et al., 2010). The thorough characterization and subsequent cloning of the essential metabolic enzymes controlling the complex homeostasis of sphingolipid components have been the focus of recent research endeavors. Their considerable influence on the molecular environment of cancer formation and the subtleties of therapy responses have been shown by this investigation (Snider et al., 2019). Recent research has illustrated the significance of sphingolipid metabolism in both lung cancer and breast cancer. Moreover, pivotal molecular targets associated with these malignancies have been pinpointed (Pei et al., 2023). In this setting, it is becoming clear that sphingolipid homeostasis disruption may play a role in the etiology of a variety of cancer morphologies, including the mysterious pancreatic adenocarcinoma. The tumor microenvironment (TME), which extends beyond the boundaries of specific cell populations, appears as a dynamic ecological niche and includes a variety of cellular and extracellular elements (Huang et al., 2023c), including tumor-associated macrophages (TAMs) (Wu et al., 2023), T-cell, and B-cell, each of which has a specific impact on the neoplastic milieu (Bejarano et al., 2021; Gong et al., 2022; Ch et al., 2023; Xiong et al., 2023). The TME is orchestrated by this complex interaction, which has a profound impact on the course of carcinogenesis and the effectiveness of treatments (Huan et al., 2023; Zhang et al., 2023j). A growing body of research has shown that the extraordinary variety within the TME supports the diverse responses seen in the context of different treatment approaches (Jin and Jin, 2020; Ng et al., 2020; Xiang et al., 2021; Chen et al., 2022).

Enrichment analyses, both using Gene Ontology (GO) and Kyoto Encyclopedia of Genes and Genomes (KEGG), have unveiled the involvement of sphingolipids in modulating the PI3K-AKT signaling pathway and T-cell proliferation pathways in the progression of PAAD. It is noteworthy that ongoing research has underscored the significant participation of sphingolipids and their associated enzymes in mediating the PI3K/AKT pathway’s influence on the growth of non-small cell lung cancer (NSCLC) cells (Gulhane and Singh, 2022). T-cell, pivotal actors in the context of NSCLC, play a vital role (Wang J. et al., 2023). These observations suggest that sphingolipids may exert their influence on the progression of PAAD by modulating the PI3K/AKT pathway and regulating T-cell activity.

Sphingolipids have been found to have a substantial role in cancer, intricately interacting with a variety of carcinogenic pathways (Ordonez et al., 2015; Liu et al., 2017; Cheng et al., 2018; Chi et al., 2022b; Liu C. et al., 2022; Kawai et al., 2022). Despite this acknowledgment, our knowledge of several genes involved in the regulation of sphingolipids is still limited, necessitating thorough exploration, particularly in light of their potential as therapeutic targets in clinical settings (Zhong et al., 2022; Zhang et al., 2023k). The cornerstone of a strong risk score signature in this study has been revealed to be a cohort of 10 genes, offering insight on their possible ramifications. In order to confirm these results, transwell tests and a wound-healing assay were carried out (Figures 9A,C,E). Given the likely function of increased SPGs expression in promoting the migratory potential of PAAD cells, our findings support the idea that sphingolipids could in fact constitute a feasible therapeutic approach for treating PAAD. The future importance of sphingolipid-focused treatments in the treatment of PAAD is thus highlighted by this study.

Changes in myelin metabolism have a significant impact on how chemosensitive neoplastic cells are, according to earlier studies. Axitinib sensitivity in people with PAAD who have elevated risk scores has been found to be particularly sensitive in the current study’s assessment of medication responsiveness (Figure 8). Using the CCK-8 test, as shown in Figure 10B, it was possible to verify the accuracy of drug predictions. The resulting results particularly highlights Axitinib’s potential eligibility as an effective treatment approach for people suffering from PAAD, particularly those who show an overexpression of SPGs (Figure 10D).

In earlier research projects, the use of gene expression profiling to classify tumor samples has been thoroughly verified (Brown and Elenitoba-Johnson, 2020; Jin et al., 2021c; Sirinukunwattana et al., 2021; Chi et al., 2022d; Jin et al., 2022). On the basis of the transcriptional levels displayed by 10 important sphingolipid-associated genes, we have attempted to classify a clinical cohort made up of PAAD patients against this scientific background. Through this methodical stratification, we have revealed striking differences in prognostic outcomes, highlighting in a profound way the strong predictive ability inherent to our genomic model, both in terms of prognosticating patient outcomes and in terms of prognosticating their responsiveness to therapeutic protocols across the spectrum from immunotherapeutic interventions to chemotherapeutic modalities. Covering the gamut from chemotherapeutic techniques to immunotherapeutic treatments. The quantity of knowledge so acquired has the potential to provide significant clinical guidance, enabling healthcare professionals to make well-informed therapy decisions for the cohort suffering from PAAD.

This study exhibits certain limitations. Despite the construction of a prognostic model based on sphingolipid-associated genes, the restricted size of the PAAD cohort hinders the extensive clinical application of this model. Furthermore, comprehensive *in vitro* experiments are imperative, whether for validating drug sensitivity or enriching the results concerning GO and KEGG signaling pathways. Predictions pertaining to immunotherapy necessitate a larger cohort of immunotherapy cases to substantiate their precision.

## 5 Conclusion

Our research efforts have resulted in the creation of a unique combination of 10 genes that make up a prognostic model. This development has the potential to help medical professionals precisely customize therapy regimens to each patient’s unique needs who has PAAD. In a related discovery, our research has shown a hitherto unknown connection between the complex environment of the immune system and the genes controlling sphingolipid metabolism. This paradigm-shifting relationship not only broadens our understanding at a fundamental level but also ushers in a new approach to immunotherapy. By focusing on the crucial sphingolipid genes, the possibility of making PAAD more susceptible to specific anti-tumor therapies emerges as an intriguing possibility deserving of future investigation.

## Data Availability

The original contributions presented in the study are included in the article/Supplementary Material, further inquiries can be directed to the corresponding author.
